# The impact of gastric adenocarcinoma location and clinical and socioeconomic determinants on survival: a retrospective population-based cohort study using SEER data (1975–2016)

**DOI:** 10.1186/s12876-026-04917-z

**Published:** 2026-05-25

**Authors:** Cynthia Tsay, Kay Chen, Laura Sahyoun, Petr Protiva

**Affiliations:** 1 Department of Medicine, Weill Cornell Medicine, Division of Gastroenterology & Hepatology, New York, NY 10065 United States; 2 Gastroenterology Section, Jennifer Moreno VA San Diego Healthcare System, San Diego, California 92161 United States; 3 Division of Gastroenterology Department of Medicine, University of California San Diego, La Jolla, San Diego, CA 92093 USA; 4 Yale School of Medicine, Section of Digestive Diseases, 333 Cedar St, New Haven, CT 06520 USA; 5 VA Connecticut Health Care System, 950 Campbell Avenue, West Haven, CT 06516 USA

**Keywords:** Gastric adenocarcinoma, Survival, Location

## Abstract

**Background:**

Despite the decreasing incidence of gastric cancer in the United States, cancers of the stomach remain one of the leading causes of cancer related death in globally. Most patients are asymptomatic and have advanced disease when diagnosed. Studies have shown similar prognosis for proximal and distal tumors, although most studies exclude lesions in the cardia given overlap with esophageal cancers. Distal tumors involving the pylorus often leads to symptoms, such as gastric outlet obstruction presenting with nausea and emesis, which may lead to earlier diagnosis; therefore, we compared the adjusted survival with gastric adenocarcinomas based on location.

**Methods:**

Using SEER*Stat software, we performed a retrospective cohort study by extracting U.S. survival data from the Surveillance, Epidemiology and End Results Database for all gastric adenocarcinomas based on location (antrum, body, fundus, pylorus) for the period between 1975 and 2016 for individuals aged > 30 years of age. Survival was compared by calculating relative hazard ratios (HRs) for cancer-specific death in the 5-year period following diagnosis with Cox proportional hazards models, adjusted for covariates, with significance set at *p* < 0.05. Data were analyzed using SAS 9.4 software and R.

**Results:**

Survival analysis included 31,158 patients and showed no survival benefit comparing adenocarcinomas of the pylorus with those in the antrum (HR 1.02, 95% CI 0.97–1.07), body (HR 1.03, 95% CI 0.97–1.09). However, lower survival was seen for those in the fundus (HR 1.19, 95% CI 1.12–1.27). Male sex (HR 1.13, 95% CI 1.10–1.27), older age (HR 1.26, 95% CI 1.21–1.30), lack of chemotherapy (HR 1.06, 95% CI 1.02–1.10) and absence of surgery (HR 1.45, 95% CI 1.38–1.53) were associated with higher mortality. There were also statistically significant differences across higher stage and grade tumors, racial groups, and marital status.

**Conclusons:**

Despite early presentation of symptoms, our study corroborated data suggesting no difference in prognosis between pyloric gastric adenocarcinomas and proximal tumors, except those in the fundus. While involvement of the pylorus often leads to clear clinical manifestations including weight loss, early satiety, nausea, and emesis, earlier identification of malignancy compared to adenocarcinomas in more indolent locations does not necessarily improve survival outcomes.

## Background

Gastric cancer is one of the most common causes of cancer and cancer-related death in the world. Across the globe, there was an estimated 1,089,103 new cases and 769,793 new deaths attributed to stomach cancer in 2020 [1]. The incidence of gastric cancers in the United States (U.S.) is lower compared to that of East Asian countries, which is likely due to differences in the rate of *Helicobacter pylori* (*H. pylori*) infection, diet, alcohol consumption and smoking [2, 3]. In addition, the incidence has been overall declining. Despite this, the estimated 5-year survival rate in the U.S. is about 32%, although this ranges from 6% to 70% depending on the stage [4]. This contrasts sharply with survival in Asia, which appears to approach 70% at 5-years [5].

Gastric cancers are often subdivided into two types, either proximal or distal gastric cancers. Proximal cancers include those located in the cardia or fundus and distal cancers include those located in the body, antrum or pylorus [6]. Tumors involving the cardia have been noted to be clinicopathologically different from those more distal and esophageal adenocarcinomas [7, 8]. Gastric cancer patients are typically asymptomatic and tend to have advanced disease at the time of diagnosis. However, distal tumors, specifically those involving the pylorus, are a common malignant cause of gastric outlet obstruction, leading to symptoms such as nausea and emesis [9]. The presence of alarm symptoms typically leads to an upper endoscopy and therefore an earlier diagnosis, compared to gastric tumors located proximally to pylorus [10].

The data on survival outcomes in patients with gastric adenocarcinoma based on the location of the primary lesion have varied. With the exclusion of tumors involving the cardia due to possible overlap with esophageal cancers, a Brazilian study [11] and Korean study [12] have demonstrated a similar prognosis for proximal and distal tumors. However, other studies that included cancers with the bulk of the tumor in the cardia with possible extension into the distal esophagus suggest that distal gastric cancers have a higher 5-year overall survival than proximal gastric cancers [13, 14]. A recent systematic review and meta-analysis showed that there was a significant difference between 5-year overall survival between proximal and distal gastric cancers, with a higher survival rate in the distal group compared to the proximal group in Eastern countries. However, this difference was not present when analyzing data from Western countries [15].

Given the conflicting results, we sought to compare the adjusted survival with gastric adenocarcinomas based on location among the U.S. population.

## Methods

The Surveillance, Epidemiology and End Results (SEER) database 18 was used to first identify all gastric cancer cases. Inclusion criteria was limited to patients with gastric adenocarcinoma by histology who were 30 years of age or older for the period between 1975 and 2016. The SEER database is comprised of 18 population-based registries which represents approximately 26% of cancer patients in the United States. Gastric adenocarcinomas were separated based on location (antrum, body, fundus, and pylorus) by using a combination of ICD-10 (C16.1, C16.2, C16.3, and C16.4) and ICD-O-3 histological codes in order to identify gastric adenocarcinomas. Exclusion criteria included tumors located in the cardia and individuals in which there was more than one primary tumor. Missing data was treated by using complete case analysis, therefore cases in which there was incomplete survival data or socioeconomic and demographic data were excluded; thus, the assumption was made that data was missing completely at random. The SEERStat software (version 8.1.5) was utilized to collect covariates of interest, which included demographic data (age, sex, race, marital status) and clinical information (date of diagnosis, primary cancer site, tumor stage and grade, survival time, and chemotherapy status). In addition, county level socio-demographic variables such as income, proportion of college level education, smoking, colorectal cancer screening, urban population, and foreign-born population were included.

We conducted a competing risk analysis using the Fine-Gray sub-distribution hazard model to evaluate the cumulative incidence of the primary event, accounting for competing events. The model was fitted using PROC PHREG in SAS. Covariate selection was guided by clinical relevance and univariate analysis. Variables with a p-value < 0.2 in univariate analysis were considered for inclusion. A forward selection process was employed, adding variables iteratively until model fit was optimized. Model assumptions were checked by plotting cumulative incidence curves for each covariate. Proportionality of sub-distribution hazards was verified visually, and time-dependent effects were assessed where necessary. The independent effect of gastric cancer location was assessed and adjusted for demographic and clinical covariates described above. Data analysis was performed using SAS 9.4 software and R, with significance set at *p* < 0.05 for the two-sided test. This study was conducted and reported in accordance with the STROBE (Strengthening the Reporting of Observational Studies in Epidemiology) recommendations to promote clear and comprehensive reporting of observational research.

## Results

**Fig. 1:**
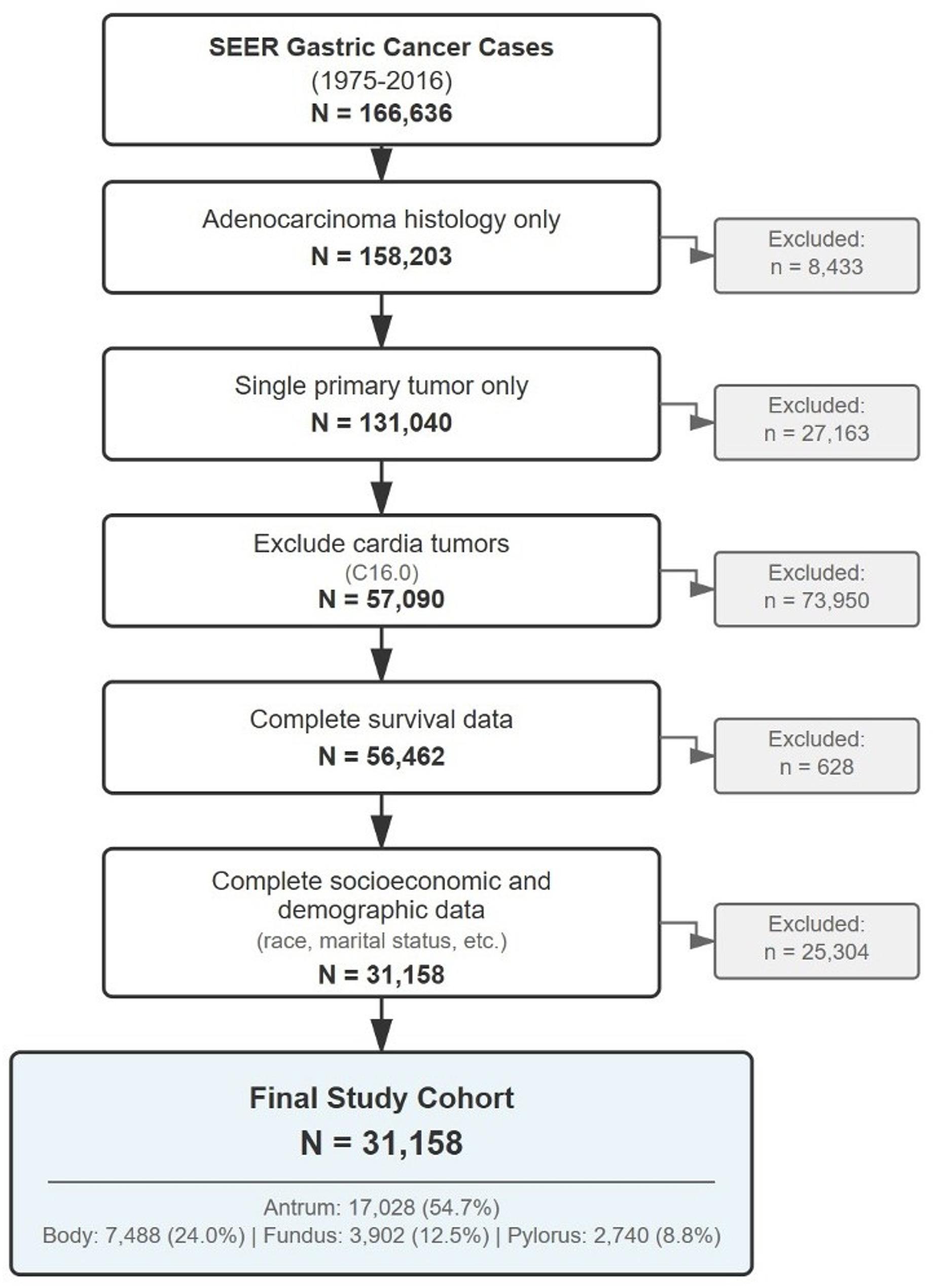
STROBE participant flow chart

We identified a total of 31,158 patients with complete survival and socio-demographic data that fit our search criteria of gastric adenocarcinoma histology with a single primary tumor location that was not located in the cardia (Fig. 1). There were 2,740 cases with gastric adenocarcinoma in the pylorus, 17,028 in the antrum, 7,488 in the body, and 3,902 in the fundus. Overall, across all locations of the gastric adenocarcinoma, there were more cases among men compared to women (antrum 55.4% versus 44.6%, body 54.4% versus 45.6%, fundus 60.1% versus 39.9%, and pylorus 56.9% versus 43.1%). The majority of the patients were also > 60 years old (antrum 78.4%, body 73.3%, fundus 71.6%, and pylorus 76.7%). While the largest percentage of patients were white, across all locations (antrum 58.1%, body 66.6%, fundus 77.1% and pylorus 63.4%), the second highest race varied based on location, with Asian or Pacific Islanders associated with higher percentages in the antrum and body compared to individuals self-identifying as Black (23.3% versus 17.8% and 18.6% versus 14.2%); compared to in the fundus (10.1% versus 11.8%) and pylorus (17.2% versus 18.0%). The majority of those identifying as non-Hispanic whites was also high compared to those identifying as Hispanic (antrum 84.1%, body 79%, fundus 83.9%, and pylorus 82.3%). Tumor stage was unknown in 33.0-44.4% of the cases, and varied between local, regional, and distant depending on anatomical location. Among the tumor grades that were known, the highest percentage of tumors among all anatomical locations were grade III tumors, followed by grade II, then grade I and grade IV tumors. The majority of patients were married across all tumor locations (antrum 52.5%, body 55.8%, fundus 55.3%, and pylorus 53.1%) with the second most common marital status across all tumors as widowed, which may be explained given the older age of the population. There was a broad general distribution temporally of when the tumors were diagnosed, although the majority seemed to have been diagnosed in the last 25 years. A minority of the patients across the tumors had documented or known chemotherapy or surgical treatment of their cancers (Table 1).

**Table 1:** Descriptive statistics (N: number of cases, % of column)

	Antrum		Body		Fundus		Pylorus	
	*N*	%	*N*	%	*N*	%	*N*	%
Sex								
Female	7602	44.6	3413	45.6	1556	39.9	1181	43.1
Male	9426	55.4	4075	54.4	2346	60.1	1559	56.9
Age								
≤ 60	3671	21.6	1999	26.7	1107	28.4	639	23.3
> 60	13,357	78.4	5489	73.3	2795	71.6	2101	76.7
Race								
American Indian/Alaskan	131	0.77	51	0.7	39	1	38	1.4
Asian or Pacific Islander	3971	23.3	1391	18.6	395	10.1	472	17.2
Black	3031	17.8	1062	14.2	459	11.8	493	18.0
White	9895	58.1	4984	66.6	3009	77.1	1737	63.4
Ethnicity								
Hispanic	2711	15.9	1575	21.0	630	16.2	486	17.7
Non-Hispanic	14,317	84.1	5913	79.0	3272	83.9	2254	82.3
Tumor Stage								
Local	3074	18.1	1893	25.3	741	19.0	381	13.9
Regional	3864	22.7	1295	17.3	550	14.1	705	25.7
Distant	3110	18.3	1830	24.4	958	24.6	438	16.0
Unknown	6980	41.0	2470	33.0	1653	42.4	1216	44.4
Tumor Grade								
Grade I	1046	6.1	719	9.6	350	9.0	200	7.3
Grade II	4713	27.7	1719	23.0	883	22.6	720	26.3
Grade III	8577	50.4	3529	47.1	1756	45.0	1290	47.1
Grade IV	300	1.8	139	1.9	84	2.2	72	2.6
Unknown	2392	14.1	1382	18.5	829	21.3	458	16.7
Marital Status								
Divorced	1315	7.7	589	7.9	366	9.4	217	7.9
Married	8946	52.5	4181	55.8	2156	55.3	1454	53.1
Single	1991	11.7	890	11.9	467	12.0	326	11.9
Unknown	704	4.1	377	5.0	166	4.3	92	3.4
Widowed	4072	23.9	1451	19.4	747	19.1	651	23.8
Year of Diagnosis								
1975–1990	3671	21.6	977	13.1	870	22.3	718	26.2
1991–2005	6605	38.8	2805	37.5	1465	37.5	1072	39.1
2006–2016	6752	39.7	3706	49.5	1567	40.2	950	34.7
Chemotherapy								
No/Unknown	12,230	71.8	5317	71.0	2609	66.9	2004	73.1
Yes	4798	28.2	2171	29.0	1293	33.1	736	26.9
Surgery								
No/Unknown	15,313	89.9	6955	92.9	3632	93.1	2405	87.8
Yes	1715	10.1	533	7.1	270	6.9	335	12.2

**Fig. 2:**
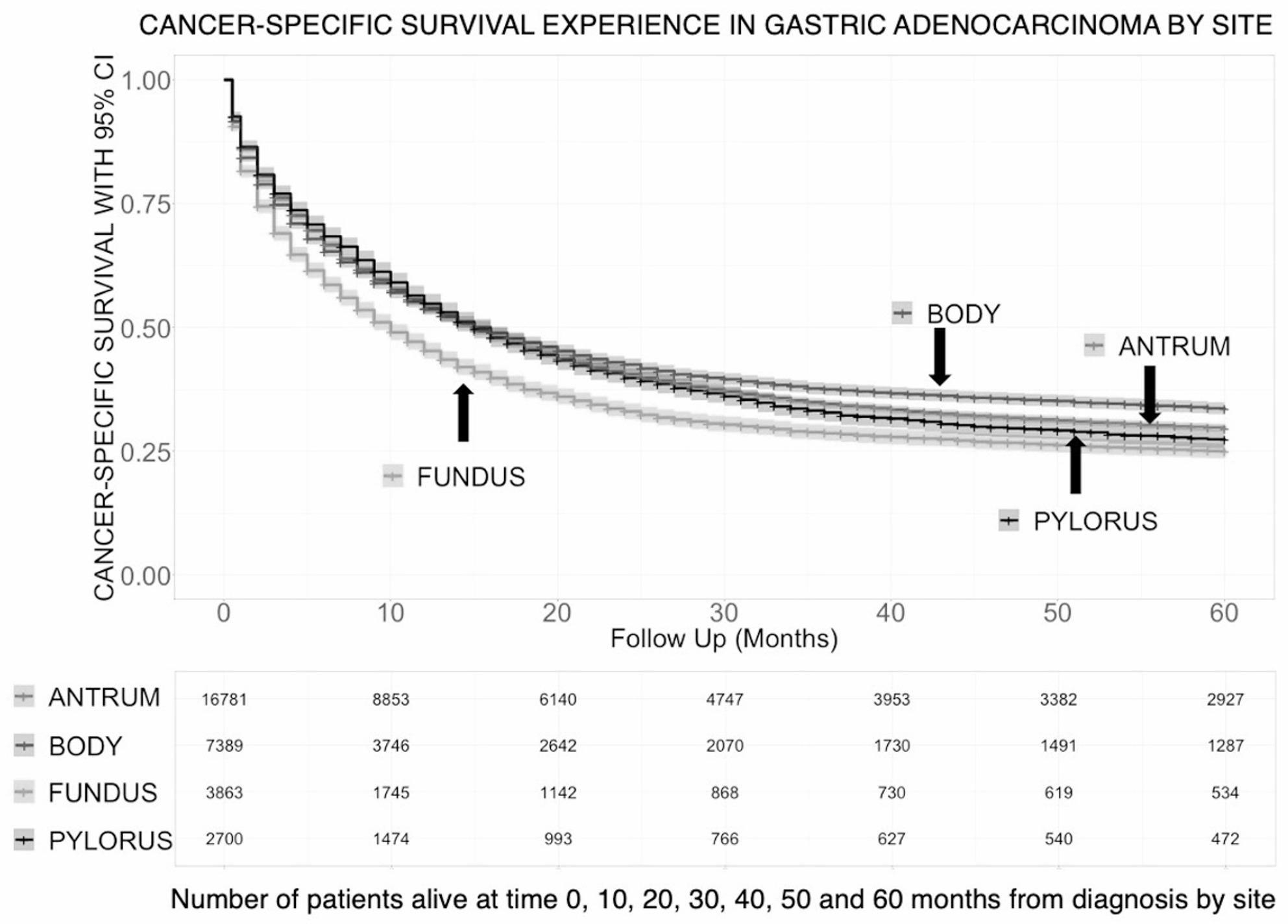
Cancer specific survival experience in gastric adenocarcinoma by primary tumor location. The Kaplan Meier survival curves are not adjusted and demonstrate that pyloric tumors are not associated with significantly better survival experience. In fact, in multivariate adjusted analysis only the tumors of fundus were associated with significantly worse survival when compared to other locations

There was no significant difference in survival experience between adenocarcinomas of the pylorus and those in the antrum (HR 1.02, 95% CI 0.97–1.07) or body (HR 1.02, 95% CI 0.97–1.08). However, higher cancer-related mortality was seen in those located in the fundus (HR 1.19, 95% CI 1.12–1.27) compared to the pylorus (Fig. 2; Table 2). As expected, cancers with a higher stage or grade were associated with higher hazard ratio for death. In addition, male sex (HR 1.19, 95% CI 1.15–1.23), age 60 or greater (HR 1.35, 95% CI 1.30–1.40), lack of chemotherapy (HR 1.13, 95% CI 1.10–1.17) and lack of surgery (HR 1.45, 95% CI 1.38–1.53) were associated with higher mortality (Table 2). The results of the adjusted Fine-Gray competing risk regression analysis for independent effect of the locations of gastric cancers and selected covariates are listed with corresponding hazard ratios and 95% confidence intervals in Table 2.

**Table 2:** Multivariable fine-gray competing risk analysis for 5-year cancer mortality

	Parameter	HR	95% CI		*P* value
Site	**Pylorus**	1			
	**Antrum**	1.02	0.97	1.07	0.542
	**Body**	1.02	0.97	1.08	0.416
	**Fundus**	1.19	1.12	1.27	< 0.0001
Sex	**Female**	1			
	**Male**	1.13	1.10	1.17	< 0.0001
Age	**< 60**	1			
	**>=60**	1.26	1.21	1.30	< 0.0001
Race	**White**	1			
	**Amer Indian/Alaska Native**	1.10	0.94	1.29	0.216
	**Asian or Pacific Islander**	0.84	0.80	0.87	< 0.0001
	**Black**	1.03	0.98	1.07	0.222
Hispanic	**No**	1			
	**Yes**	0.95	0.91	0.99	0.011
Stage	**Local**	1			
	**Distant**	7.26	6.82	7.73	< 0.0001
	**Regional**	2.79	2.62	2.97	< 0.0001
	**Unknown**	3.28	3.08	3.49	< 0.0001
Grade	**I: Well differentiated**	1			
	**II: Moderate differentiated**	1.62	1.50	1.75	< 0.0001
	**III: Poorly differentiated**	2.11	1.96	2.28	< 0.0001
	**IV: Undiff/anaplastic**	2.23	1.98	2.51	< 0.0001
	**Unknown**	1.64	1.52	1.78	< 0.0001
Marital Status	**Married**	1			
	**Divorced**	1.08	1.03	1.14	0.0034
	**Single (never married)**	1.10	1.05	1.15	0.0001
	**Widowed**	1.18	1.14	1.23	< 0.0001
	**Unknown**	0.97	0.90	1.05	0.491
Year of Diagnosis	**1975–1990**	1			
	**1991–2005**	0.98	0.93	1.03	0.389
	**2006–2016**	0.85	0.80	0.90	< 0.0001
Chemotherapy	**Yes**	1			
	**No/Unknown**	1.06	1.02	1.10	0.0008
Surgery	**Yes**	1			
	**No/Unknown**	1.45	1.38	1.53	< 0.0001
Colorectal Cancer Screening	**Below median**	1			
	**Above median**	0.94	0.91	0.97	< 0.0001
Urban Location	**Below median**	1			
	**Above median**	0.94	0.90	0.97	0.0001
Smokers (%)	**Below median**	1			
	**Above median**	0.99	0.96	1.04	0.967
College Graduation (%)	**Below median**	1			
	**Above median**	1.01	0.97	1.05	0.692
Income Level	**Below median**	1			
	**Above median**	0.98	0.94	1.02	0.266
Foreign Birth (%)	**Below median**	1			
	**Above median**	0.98	0.94	1.03	0.462

Statistically significant differences were also seen across racial and ethnic groups and marital status. Black patients had a similar mortality (HR 1.03, 95% CI 0.98–1.07) when compared to the white reference group, whereas Asian and Pacific Islanders had a lower mortality (HR 0.84, 95% CI 0.80–0.87). Patients of Hispanic ethnicity were also noted to have a lower mortality (HR 0.95, 95% CI 0.91–0.99). In terms of marital status, being divorced (HR 1.08, 95% CI 1.03–1.14), single (HR 1.10, 95% CI 1.05–1.15), or widowed (HR 1.18, 95% CI 1.14–1.23) was associated with a higher mortality than being married. Living in a highly urban area or an area with high levels of colorectal screening was associated with increased survival (HR 0.94, 95% CI 0.90–0.97, HR 0.94, 95% CI 0.91–0.97, respectively). There was no significant difference in cancer-related mortality in regards to level of education, smoking status, income level, or foreign location of birth.

## Discussion

Our study corroborated data suggesting no difference in prognosis between gastric adenocarcinomas involving the pylorus compared to those in the antrum and the body, even though pyloric involvement may lead to an earlier diagnosis due to the presence of symptoms. Interestingly, our study found that there was a higher cancer-related mortality for lesions located in the fundus compared to the pylorus, which should warrant future investigation.

The literature remains varied with respect to whether the location of the primary tumor impacts overall survival. The variability in findings may be related to the ways proximal and distal tumors are classified. For example, some studies have included tumors involving the cardia, which can be secondary to esophageal cancer. In some instances, there may be proximal migration of the tumor from the initial location, which would affect classification. However, as noted earlier, the epidemiology of gastric cancers influences survival outcomes. There are many factors which may explain our findings of higher cancer-related mortality for lesions in the fundus compared to the pylorus, including tumor stage and grade at diagnosis, tumor biology, differing *H. pylori* strains, the approach to surgical resection and lymphadenectomy, and treatment strategies [14–16]. Additionally, research suggests that while *H. pylori* often adheres to the antrum, with the widespread use of proton pump inhibitors, migration of the bacteria to the gastric body in those on chronic acid suppression therapy may be a risk factor for gastric cancer [17, 18].

Other factors that may affect prognosis include different molecular subtypes of gastric cancer, as these are associated with varying tumor locations and response to chemotherapy [19, 20]. Genomic differences in the tumor, such as differences in large sequences of b-cadherin in Caucasian compared to black or Asian patients or variability of vascular endothelial factor expression in Caucasian and Asian patients, may or may not lead to prognostic differences as well [21, 22]. Clinicopathological differences have previously been described, with gastric cancers of the cardia possibly being more aggressive due to higher lymphovascular invasion, esophageal invasion, and histological transformation from differentiated to undifferentiated tumor type, compared to distal cancers [7]. Additional research needs to be done to explore these differences.

In terms of other factors that affect survival, our study demonstrated similar survival among blacks compared to whites (although the HR was 1.05, the 95% CI included the null when rounded), unlike other studies which have shown higher mortality among blacks [23–25]. We also confirmed that being of Asian / Pacific Islander descent is associated with a lower mortality. This has been found to be an independent predictor of survival in multiple studies, irrespective of adjuvant treatment or socioeconomic status, and this is thought to be related to earlier age at diagnosis [23, 24]. This may be in part related to screening protocols with endoscopy in Asian immigrants, leading to earlier detection and treatment. In addition, being of Hispanic ethnicity is associated with improved survival, despite the higher incidence of early onset gastric cancer and higher presence of disadvantageous socioeconomic factors. This has been seen in other studies as well and suggests a biological difference in this population [26–28]. A 2006 review of studies preceding the start of our dataset suggested a possible demographic association with proximal cardia tumors and older white men and with distal tumors and black individuals. However, the changing epidemiology of gastric cancer and our study results did not corroborate this finding [29]. Our study also shows that living in an urban location and having high prevalence of CRC screening is associated with improved survival, suggesting that county level attributes could serve as proxy for access to healthcare. Concordantly, a recent study suggested better survival outcomes for individuals with gastric adenocarcinoma treated at academic research hospitals compared to non-academic hospitals, with multivariable Cox regression demonstrating survival benefit even when adjusting for sociodemographic factors and hospital volume [30]. This suggests that access to tertiary care centers that can offer novel treatments may impact survival among patients with gastric adenocarcinoma. Lastly, the positive effect of marriage on survival outcomes has been shown previously and is further supported by our study [31–33]; this is possibly related to increased social and psychological support.

## Limitations of the study

At present, this is the largest comparative analysis looking at the location of gastric adenocarcinoma and its effects on prognosis. However, there were some limitations. Firstly, the SEER database only contains information about cancer patients within the U.S., so the results may not translate globally. Secondly, to protect privacy, several socio-demographic variables are derived using county attributes and not individual data. Thirdly, as with all registry data, the number and granularity of available variables are limited. This limits the ability to assess outcomes outside of mortality due to the lack of data captured on recurrence or progression, as well as the ability to select for prognostic factors that may influence overall survival and to identify confounding factors such as *H. pylori* or Epstein-Barr virus status. The presence of endoscopic or histologic findings that are suggestive of an increased risk for gastric cancer were not considered. Additionally, some variables were nominal categorical variables, which can introduce misclassification and residual confounding factors. Fourthly, tumor location is registry-coded without centralized pathology review, which can lead to misclassification around anatomical boundaries. Lastly, as with any observational study, unmeasured and residual confounding factors cannot be fully excluded.

## Conclusion

Therefore, while involvement of the pylorus often leads to clear clinical manifestations including weight loss, early satiety, nausea, and emesis, our study suggests that earlier identification of malignancy compared to adenocarcinomas in more indolent locations of the stomach does not necessarily improve survival outcomes.

## Data Availability

The dataset supporting the conclusions of this article is available in the National Cancer Institute Surveillance, Epidemiology and End Results database to those who request access via: https://seer.cancer.gov/data/.

## Abbreviations

U.S.: United States
SEER: Surveillance, Epidemiology, and End Results
HR(s): Hazard ratio(s)
